# Observation of the effectiveness of clinical indicators of cardiac shock wave therapy in patients with ischemic heart disease: A systematic review and meta-analysis

**DOI:** 10.3389/fcvm.2023.1088811

**Published:** 2023-01-24

**Authors:** Xinze Wu, Minghong Gu, Yixuan Ma, Peiyu Song, Chenghu Fang

**Affiliations:** ^1^Department of Internal Medicine and Rehabilitation Science, Tohoku University Graduate School of Medicine, Tohoku University, Sendai, Japan; ^2^Department of Pain Management, Jiangwan Hospital of Shanghai Hongkou District, Shanghai University of Medicine and Health Science Affiliated First Rehabilitation Hospital, Shanghai, China; ^3^Division of Sports Science and Physical Education, Tsinghua University, Beijing, China; ^4^Department of Cardiology, Jiangwan Hospital of Shanghai Hongkou District, Shanghai University of Medicine and Health Science Affiliated First Rehabilitation Hospital, Shanghai, China

**Keywords:** ischemic heart disease, cardiac shock wave therapy, cardiac function, meta-analysis, review

## Abstract

**Objective:**

Ischemic heart disease (IHD) has a high prevalence and mortality rate, imposing a heavy burden on patients and society, and there is still a need to optimize treatment options for IHD patients. Cardiac shock wave therapy (CSWT) is gaining popularity as a new treatment for IHD patients. The objective of this meta-analysis is to reassess the effects of CSWT on IHD patients in light of the limited number of clinical studies included in previously published reviews, inconsistent methodological quality, and unclear outcomes.

**Methods:**

From database creation until September 1, 2022, 4 English databases and 3 Chinese databases were rigorously searched for any current controlled trials of CSWT for IHD. The Cochrane Risk of Bias Assessment Tool was used for methodological quality assessment. Review Manager v.5.4 software was used for meta-analysis.

**Results:**

Nineteen published controlled trials totaling 1,254 subjects were included. Results showed that CSWT could enhance left ventricular function and myocardial viability, improve cardiac function and alleviate angina pectoris symptoms. The effects of CSWT and control groups on SAQ scores and exercise time were not statistically significant.

**Conclusion:**

According to this systematic review and meta-analysis, CSWT may be beneficial for a number of IHD clinical indications. To verify these findings, more RCT studies with bigger sample numbers and higher methodological standards are required in the future.

## 1. Introduction

Since 1990, 9.14 million deaths have been attributed to ischemic heart disease (IHD) until 2019 when the prevalence of IHD is as high as 197 million cases. The healthcare burden due to IHD is steadily increasing (1). Despite improvements in prevention and treatment for IHD over the past several years in clinical practice procedures, the mortality and morbidity associated with this disorder still place a major burden on human health (2, 3).

Currently available treatments include medication therapy, percutaneous coronary intervention (PCI), and coronary artery bypass grafting (CABG) (4, 5). Several studies have investigated some new alternative treatment options for refractory angina, such as stem cell therapy, trans myocardial revascularization, and percutaneous myocardial laser revascularization. However, the majority of these treatments are invasive and still in the early stages of development (6).

Cardiac shock wave therapy (CSWT) is a brand-new non-invasive technique that utilizes shockwaves to target and concentrate distinctive acoustic waves on a particular region of the heart while using echocardiography as guidance. This treatment may promote myocardial perfusion and decrease symptoms of myocardial ischemia (7). One of its more intriguing uses in cardiovascular medicine is as a potential treatment for individuals with refractory angina due to the reported instantaneous increase in blood flow brought on by local vasodilation and the emergence of new capillaries in the treated tissue (7). Currently, animal studies have demonstrated that CSWT can improve left ventricular remodeling (8), promote cardiomyocyte survival (9), and has anti-inflammatory effects (10). However, there are still relatively few trials for clinical purposes and the sample sizes included in trials are generally small. The determination of the effectiveness of CSWT in humans remains to be studied.

The objective of this meta-analysis is to reevaluate the effects of CSWT in patients with IHD in light of the few clinical studies that were included in prior published reviews, the mixed and unclear outcome and the inconsistent methodological quality of the published research.

## 2. Materials and methods

The Preferred Reporting Items for Systemic Review and Meta-Analyses (PRISMA) and the Cochrane Handbook for Systematic Reviews of Interventions were used in the design of this study (11).

### 2.1. Search strategy

From initial establishment until September 1, 2022, the following electronic databases were searched: PubMed, Excerpta Medica database (EMBASE), Web of Science and Cochrane Library (for relevant English academic paper), and China National Knowledge Infrastructure (CNKI), Wan Fang Database, and China Science and Technology Journal (VIP) (for relevant Chinese academic paper). The following search tactics were combined: for English databases, the terminologies were “cardiac shock wave therapy” OR “CSWT” OR “ESWT” AND “myocardial ischemia” OR “angina” OR “ischemic heart disease” were adopted, and in the Chinese databases, the terminologies were “xin zang zhen bo zhi liao” AND “que xue xing xin zang bing” OR “xin jiao tong” OR “xin ji que xue”. For this meta-analysis, only free publications in Chinese and English were included in the analysis.

### 2.2. Study selection

The following criteria had to be met in order for a study to be included: (1) having a population of participants with IHD; (2) using any type of cardiac shock wave therapy as an intervention (high or low intensity treatment); (3) having a control group, not use a comparison of one’s own condition before and after treatment; or (4) reporting results of symptoms associated with IHD. The following were the exclusion criteria: (1) not in English or Chinese; (2) lack of data or results that aren’t really relevant; (3) no access to the entire text.

### 2.3. Data extraction and quality assessment

Two unbiased reviewers independently extracted the data and evaluated all the studies in accordance with the predetermined standards (YM and PS). Data were checked by the third reviewer (XW). Authors’ name, publication year, number of cases, mean age of participants, treatment methods (type of treatments, control details, and duration of treatment) were among the details extracted from the included studies.

Using the risk of bias tool from the Cochrane Collaboration, we evaluated the quality of the included studies. Each item accustomed one of three array (low risk, uncertain, and high risk) based on the afterward standards: the conception of accidental sequences, the beard of allocations, the blinding of participants and staff, the bare after effect data, the careful reporting, and any added biases.

### 2.4. Statistical analysis

Applying the program Review Manager v.5.4 software, all data and statistical analyses were fully integrated (Cochrane Collaboration, Oxford, UK). To examine continuous results, the mean difference (MD) and 95% confidence intervals (CIs) were used. The mean and standard deviation of the two combinations were calculated according to the method described in Chapter 6 6.5.2.10 of the Cochrane Handbook, 6th Edition (2019). Heterogeneity between studies was tested by chi-square test and Higgins *I*^2^ statistical (12). When statistical heterogeneity was low (*I*^2^ ≤ 50% or Chi^2^ test *P* < 0.10), a fixed effect model was used. In addition, a random effects model was used (12). Potential sources of heterogeneity were identified using a sensitivity analysis that examined the effect of sequentially removing each study on the pooled estimates. Statistics were considered significant at a *P*-value of 0.05 or below. The regression asymmetry test by Egger was used to identify publication bias.

## 3. Results

### 3.1. Study selection and the basic information

**FIGURE 1:**
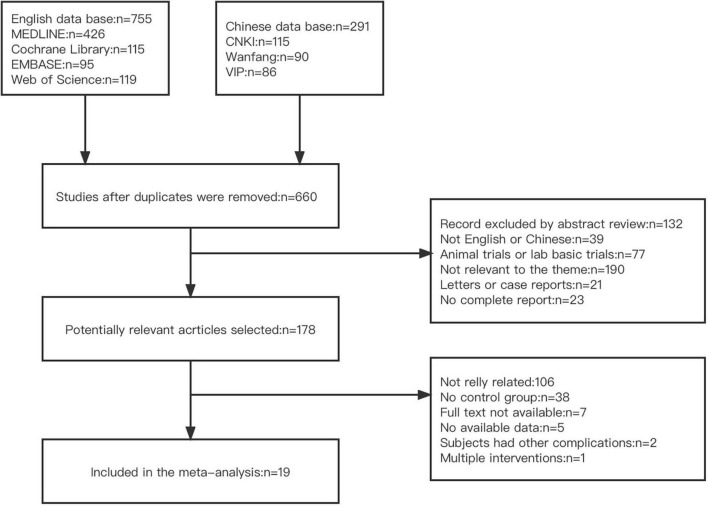
Flow diagram of screening literature studies.

There were 1,046 studies found in total after database scanning (755 English literature studies and 291 Chinese literature studies). Six hundred and sixty studies were evaluated after duplicate submissions were removed. According to the article abstract and title, 482 studies were excluded. One hundred and seventy-eight full-text analysis of potentially relevant studies was identified. One hundred and fifty-nine studies were excluded because of (1) not really related; (2) not set a control group; (3) not available full text; (4) no available data; (5) subjects had other complications and (6) intervention overlapped with other methods. A total of 19 studies were included following screening, Figure 1 depicts this study selection’s flow chart.

**FIGURE 2:**
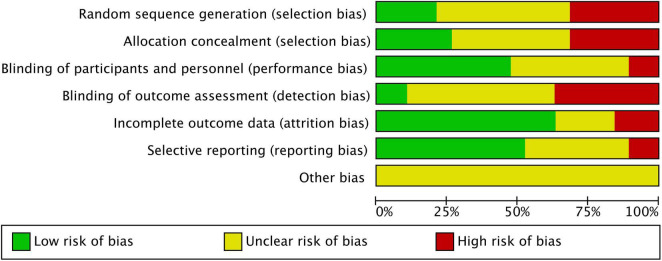
Risk of bias graph.

**FIGURE 3:**
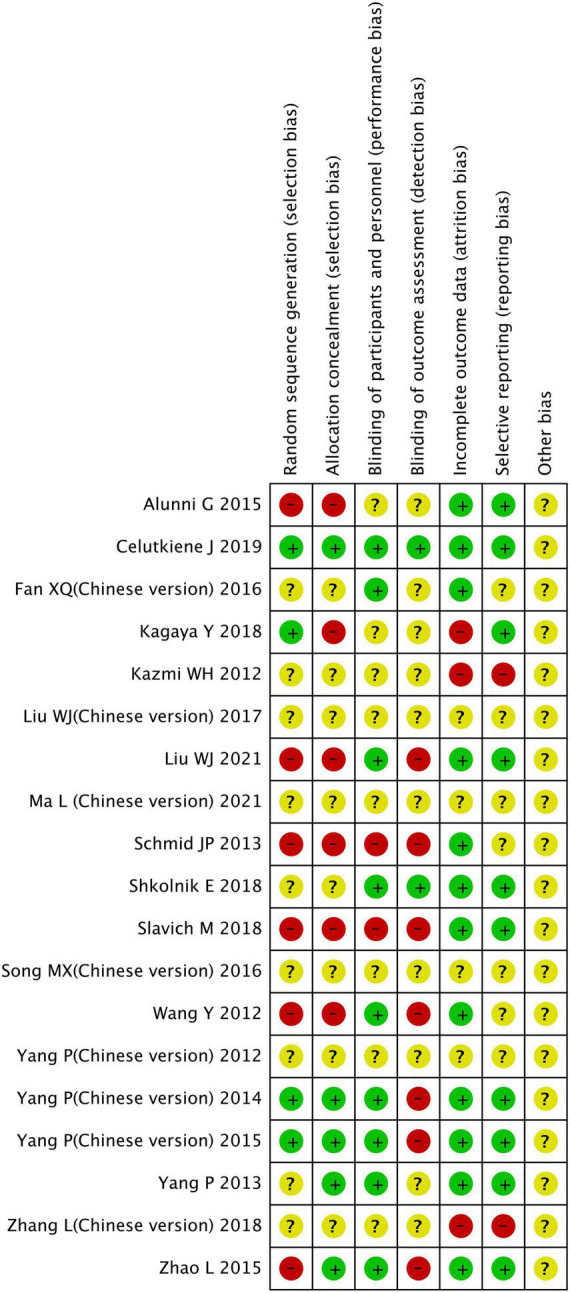
Risk of bias summary.

This study included 19 clinical studies and a add up to 1,254 patients, including 726 patients treated with CSWT and 528 patients were the control group. 12 months was the longest observation period. Table 1 displays the main characteristics of all included research. Figures 2, 3 appearance the affection appraisal of the included studies application Cochrane Collaboration.

**TABLE 1:** Characteristics of trials included in the meta-analysis.

Trials	Alunni et al. (7)	Celutkiene et al. (33)	Fan et al. (19) (Chinese version)	Kagaya et al. (16)	Kazmi et al. (15)	Liu et al. (34) (Chinese version)	Weijing et al. (6)	Ma et al. (17) (Chinese version)	Schmid et al. (35)	Shkolnik et al. (36)
Participates	72	59	68	32	86	45	87	70	21	72
Target population	Refractory angina	Stable angina	CAD	Acute myocardial infarction	End stage CAD	Stable angina	Chronic refractory angina pectoris	CAD	Chronic refractory angina pectoris and myocardial ischemia	Stable angina
Study design	Case–control study	RCT	RCT	Historical control study	Prospective cohort study	RCT	RCT	RCT	RCT	RCT
Follow-up	6 m	6 m	3 m	12 m	6 m	Unclear	6 m	3 m	3 m	6 m
Age (CSWT/control)	70 ± 5.3/71 ± 5.3	67.2 ± 7.8/69.4 ± 7.8	69.8 ± 12.0/67.2 ± 10.0	65.0 ± 7.3/67.3 ± 12.8	58.7 ± 9.5/56.6 ± 11.6	70.5 ± 5.6/65.9 ± 8.2	68.1 ± 6.7/68.9 ± 6.6	65.83 ± 6.3/64.4 ± 6.7	72.6 ± 8.3/63.4 ± 3.2	67.6 ± 8.3/68.8 ± 8.3
BMI (CSWT/control)	/	30.0 ± 4.3/30.3 ± 3.8	22.8 ± 2.2/21.7 ± 3.2	/	/	26.4 ± 2.6/27.6 ± 2.9	24.7 ± 3.8/24.9 ± 3.7	25.93 ± 2.4/25.3 ± 2.5	/	29.7 ± 4.1/30.1 ± 3.8
Male	60	45	30	27	74	31	61	53	19	51
Smoke (actual or prior)	/	5	23	/	/	16	31	25	6	8
Hypertension	72	58	/	/	43	26	50	45	16	70
Hyperlipidemia	69	59	/	/	/	20	38	43	18	61
Diabetes	22	16	19	/	65	24	47	23	4	18
PCI	59	31	/	/	23	/	/	/	/	38
CABG	30	38	/	/	61	/	/	/	13	40
Beta blockers	65	55	19	25	/	24	47	63	15	/
Calcium channel blocker	/	31	20	/	/	15	29	18	7	/
ACEI/ARB	/	59	24	32	/	/	40	66	17	/
Aspirin	68	/	52	/	/	40	77	66	/	/
Statins	66	59	/	29	/	/	/	64	19	/
Chronic therapy with nitrates	51	34	30	/	/	/	/	41	10	/
Antiplatelet agents	29	59	/	/	/	/	/	/	21	/
Participates	23	53	55	45	25	87	87	180	87	
Target population	Refractory angina	Ischemic heart disease	Severe CAD	CAD	CAD	CAD	Old myocardial infarction (OMI)	CAD	Old myocardial infarction (OMI)	
Study design	Retrospective study	RCT	RCT	RCT	RCT	RCT	RCT	RCT	RCT	
Follow-up	Unclear	3 m	12 m	6 m	6 m	12 m	12 m	Unclear	12 m	
Age (CSWT/control)	69.79 ± 10.22/65.25 ± 5.74	67 ± 6/66 ± 7	63.4 ± 10.8/67.9 ± 7.8	67.5 ± 9.4/66.1 ± 9.7	63.71 ± 8.60/66.45 ± 8.51	67.03 ± 8.57/66.24 ± 8.14	67.03 ± 8.57/66.24 ± 8.14	62.5 ± 6.8/61.3 ± 7.2	67.03 ± 8.57/66.24 ± 8.14	
BMI (CSWT/control)	/	/	23.9 ± 2.8/24.0 ± 3.2	23.5 ± 1.3/22.8 ± 1.6	/	23.76 ± 1.78/23.01 ± 1.65	/	/	23.76 ± 1.78/23.01 ± 1.65	
Male	18	22	47	36	18	48	48	100	68	
Smoke (actual or prior)	16	20	22	12	13	26	/	/	26	
Hypertension	19	32	39	32	13	55	/	/	55	
Hyperlipidemia	19	/	14	28	16	45	/	/	45	
Diabetes	11	23	15	19	11	39	/	/	39	
PCI	/	/	/	30	/	/	/	/	/	
CABG	/	/	/	8	/	/	/	/	/	
Beta blockers	20	/	49	34	16	64	/	/	64	
Calcium channel blocker	8	/	21	27	12	45	/	/	45	
ACEI/ARB	/	/	31	30	17	57	/	/	57	
Aspirin	/	/	48	35	17	61	/	/	61	
Statins	/	/	48	37	18	58	/	/	58	
Chronic therapy with nitrates	13	/	21	34	15	65	/	/	65	
Antiplatelet agents	/	/	34	26	/	34	/	/	34	

The study’s final observation indexes were as follows: (1) left ventricular ejection fraction (LVEF); (2) left ventricular end diastolic dimension (LVEDD); (3) New York Heart Association (NYHA) class; (4) Time required for 6 min walking test (6MWT); (5) Seattle Angina Questionnaire score (SAQ); (6) Canadian Cardiology Society angina class (CCS); (7) Exercise duration (min); (8) Total score of perfusion imaging; (9) Total score of metabolism imaging; (10) Nitrate consumption (times/week).

### 3.2. Sensitivity analysis

To investigate potential sources of variability, sensitivity analyses were conducted based on removing studies with low quality, small sample numbers, and the result of research with extremely abnormal data. A distinct sensitivity analysis was carried out for each of these substantially varied results.

### 3.3. Results of the indicator analyses

#### 3.3.1. LVEF

**FIGURE 4:**
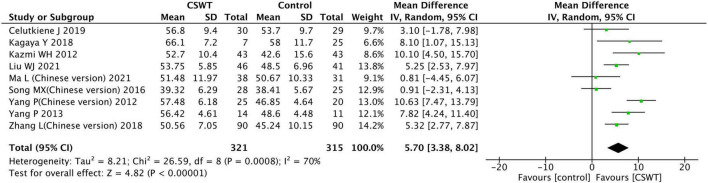
Forest plot of the CSWT group vs. the control group-LVEF.

**FIGURE 5:**
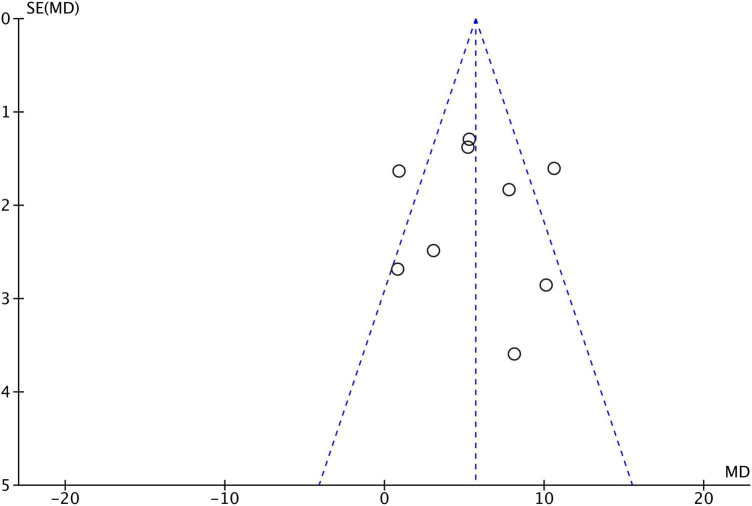
Funnel plot of publication bias.

Nine studies reported the LVEF in the CSWT and control groups after the experiment, with a sum of 636 patients. The included research types were 7 RCT studies, 1 prospective cohort study, and 1 historical control study (Figure 4). The heterogeneity results indicated that there was heterogeneity in each trial (*P* < 0.001, *I*^2^ = 70%), which were carried out with the aid of a random effects model. According to the results of the observation, there was a significant difference between the CSWT and control groups (MD 5.70, 95% CI 3.38∼8.02, *P* < 0.001). Furthermore, no significant publication bias was discovered in the funnel plot of the nine research (Figure 5).

The sensitivity analyses were performed. Firstly, 3 studies with low quality were excluded (13–15). The aggregated data indicated that this exclusion had no appreciable impact on the outcomes, and the heterogeneity analysis indicated that there was no heterogeneity (*P* = 0.30, *I*^2^ = 18%), and performed using a fixed effects model statistical. The CSWT and control groups were significantly different (MD 5.29, 95% CI 3.82∼6.76, *P* < 0.001). Secondly, 1 study (16) because of a small sample size excluded. However, this exclusion had no substantial impact on statistical heterogeneity (*P* = 0.24, *I*^2^ = 27%) and results (MD 5.16, 95% CI 3.66∼6.66, *P* < 0.001), which were conducted using a fixed effects model. The results of all sensitivity studies were consistent, indicating the stability of the findings reported here.

#### 3.3.2. LVEDD

**FIGURE 6:**
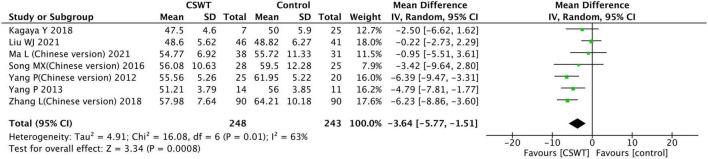
Forest plot of the CSWT group vs. the control group-LVEDD.

Seven studies reported the LVEDD of the CSWT group and the control group after the experiment, with a total of 491 patients. The included study types were 6 RCT studies and 1 prospective cohort study (Figure 6). Results of the heterogeneity analysis indicated that there was heterogeneity in each study (*P* = 0.010, *I*^2^ = 63%), which was conducted using a random effects model. The findings indicate that there was a significant difference between the CSWT and control groups (MD −3.64, 95% CI −5.77∼−1.51, *P* < 0.001).

The sensitivity analyses were performed. Two studies with low quality were excluded (13, 17). The aggregated data indicated that this exclusion had no appreciable impact on the outcomes, and the heterogeneity analysis indicated that there was a heterogeneity (*P* = 0.020, *I*^2^ = 65%), and performed using a random effects model statistical. There was a significant difference between the CSWT and control groups (MD −3.45, 95% CI −6.02∼−0.88, *P* < 0.001). Then, 1 study (16) because of a small sample size excluded, but the statistical heterogeneity was not significantly altered by this exclusion (*P* = 0.010, *I*^2^ = 74%) which was also conducted using a random effects model, and results were not materially altered (MD −3.66, 95% CI −6.81∼−0.52, *P* = 0.020). The results of all sensitivity analysis were consistent, indicating the stability of the findings reported here.

#### 3.3.3. NYHA class

**FIGURE 7:**
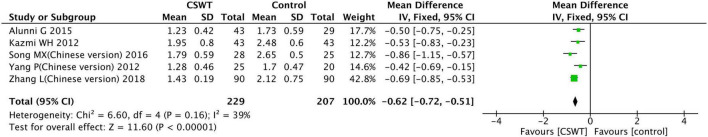
Forest plot of the CSWT group vs. the control group-NYHA class.

Eight studies reported the NYHA class of the CSWT group and the control group after the experiment, but three studies (18–20) reported results using quartiles and were not included in the analysis. Five studies were included in the final analysis, with a total of 436 patients. Among the studies included in the analysis, there were 3 RCT studies, 1 case-control study, and 1 prospective cohort study (Figure 7). The findings of the heterogeneity analysis indicated that there was none in each trial (*P* = 0.016, *I*^2^ = 39%), and conducted using a fixed effects model. The results suggest that there was a significant difference between the CSWT and control groups (MD −0.62, 95% CI −0.72∼−0.51, *P* < 0.001).

#### 3.3.4. 6MWT

**FIGURE 8:**
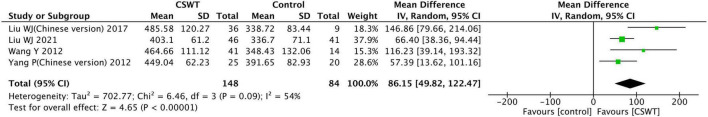
Forest plot of the CSWT group vs. the control group-6MWT.

Six studies reported the 6MWT of the CSWT group and the control group after the experiment, but two studies (18, 19) reported results using quartiles and not included in the analysis. Four studies were included in the final analysis, with a total of 232 patients, these all were RCT studies (Figure 8). The heterogeneity results indicated that there was heterogeneity in each trial (*P* = 0.090, *I*^2^ = 54%), which were conducted using a random effects model. The findings indicate that there was a significant difference between the CSWT and control groups (MD 86.15, 95% CI 49.82∼122.47, *P* < 0.001).

#### 3.3.5. SAQ score

**FIGURE 9:**
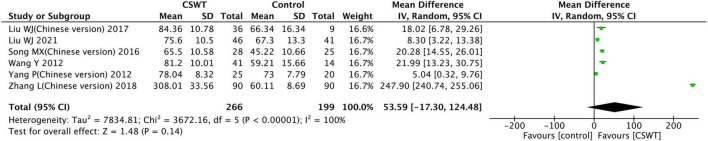
Forest plot of the CSWT group vs. the control group-SAQ score.

Eight studies reported the SAQ total score of the CSWT group and the control group after the experiment, but two studies (18, 19) reported results using quartiles and so were not included in the analysis. Six studies were included in the final analysis, with a total of 465 patients, these all were RCT studies (Figure 9). The heterogeneity results indicated that there was heterogeneity in each trial (*P* < 0.001, *I*^2^ = 100%), and conducted using a random effects model. The findings suggest that the CSWT and control groups did not have a significant difference (MD 53.59, 95% CI −17.30∼124.48, *P* = 0.140).

One study (21) with outliers was excluded. The aggregated results indicate that this exclusion significantly altered the findings, and the heterogeneity analysis showed that there was a heterogeneity (*P* < 0.001, *I*^2^ = 84%), and performed using a random effects model statistical. The CSWT and control groups were significantly different (MD 14.14, 95% CI 6.99∼21.29, *P* = 0.0001). Results from analyses of this indicator were inconsistent.

#### 3.3.6. CCS class

Eleven studies reported the CCS class of the CSWT group and the control group after the experiment, but three studies (18–20) reported results using quartiles and not included in the analysis.

**FIGURE 10:**
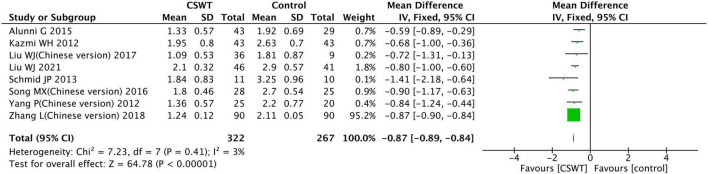
Forest plot of the CSWT group vs. the control group-CCS class.

Eight studies were included in the final analysis, with a total of 589 patients. The included study types were 6 RCT studies, 1 case-control studies and 1 prospective cohort study (Figure 10). According to heterogeneity results, none of the studies were homogeneous (*P* = 0.410, *I*^2^ = 3%), and conducted using a fixed effects model. The findings indicate that the CSWT and control groups differed significantly (MD −0.87, 95% CI −0.89∼−0.84, *P* < 0.001).

#### 3.3.7. Exercise duration

**FIGURE 11:**
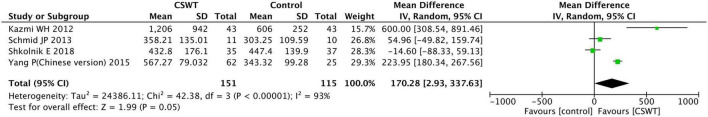
Forest plot of the CSWT group vs. the control group-exercise duration.

Four studies reported the exercise duration of the CSWT group and the control group after the experiment, with a total of 266 patients. The included study types were 3 RCT studies, and 1 prospective cohort study (Figure 11). According to heterogeneity results, each study was homogeneous (*P* < 0.001, *I*^2^ = 93%), and conducted using a random effects model. The results suggest that there was no significant difference between the CSWT and control groups (MD 170.28, 95% CI 2.93∼337.63, *P* = 0.050).

One study had significantly higher data than the others, and no significant changes were observed when that study was excluded. And the heterogeneity analysis showed that there was a heterogeneity (*P* < 0.001, *I*^2^ = 94%), conducted using a random effects model. The findings suggest that the CSWT and control groups did not differ significantly (MD 92.80, 95% CI −76.25∼257.89, *P* = 0.290).

#### 3.3.8. Total score of perfusion imaging

**FIGURE 12:**
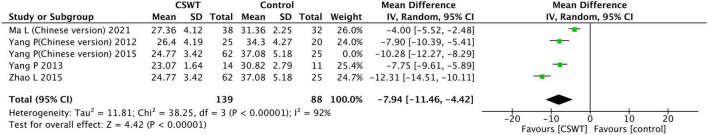
Forest plot of the CSWT group vs. the control group-total score of perfusion imaging.

Six studies reported the total score of perfusion imaging of the CSWT group and the control group after the experiment, we found that two studies reported the same value with different languages (22, 23), 1 study was excluded (23). One study (19) reported results using quartiles and were not included in the analysis. Four studies were included in the final analysis, with a total of 227 patients, and these all were RCT studies (Figure 12). According to heterogeneity results, each study was homogeneous (*P* < 0.001, *I*^2^ = 92%), and conducted using a random effects model. The findings indicate that the CSWT and control groups differed significantly (MD −7.94, 95% CI −11.46∼−4.42, *P* < 0.001).

#### 3.3.9. Total score of metabolism imaging

**FIGURE 13:**
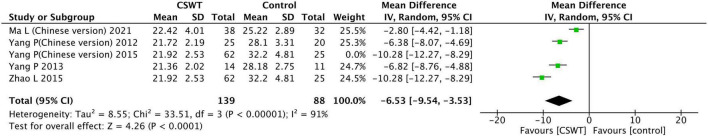
Forest plot of the CSWT group vs. the control group-total score of metabolism imaging.

Six studies reported the total score of metabolism imaging of the CSWT group and the control group after the experiment, we found that two studies reported the same value with different languages (22, 23), 1 study was excluded (23). One study (19) reported results using quartiles and were not included in the analysis. Four studies were included in the final analysis, with a total of 227 patients, and these all were RCT studies (Figure 13). According to heterogeneity results, each study was homogeneous (*P* < 0.001, *I*^2^ = 91%), and conducted using a random effects model. The findings indicate that the CSWT and control groups differed significantly (MD −6.53, 95% CI −9.54∼−3.53, *P* < 0.001).

#### 3.3.10. Nitrate consumption (times/week)

**FIGURE 14:**
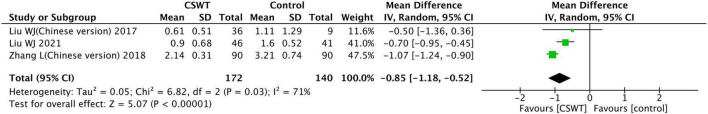
Forest plot of the CSWT group vs. the control group-nitrate consumption (times/week).

Three studies reported the exercise duration of the CSWT group and the control group after the experiment, with a total of 312 patients. These all were RCT studies (Figure 14). According to heterogeneity results, each study was homogeneous (*P* = 0.003, *I*^2^ = 71%), and conducted using a random effects model. The findings indicate that the CSWT and control groups differed significantly (MD −0.85, 95% CI −1.18∼−0.52, *P* < 0.001).

## 4. Discussion

This study is a meta-analysis that attempts to assess the impact of CSWT in patients with IHD, we included the most recent literature and analyzed a variety of clinical indicators.

A major advantage of CSWT over PCI, CABG, and transmyocardial laser revascularization is shown by the fact that it is quite non-invasive and safe, without any procedural complications or adverse effects (5). A high-frequency, low-energy electromagnetic ultrasonic pulse known as the CSWT treatment system has the ability to instantly produce extremely high-pressure sound waves. After interstitial reflection, the pulse wave is finely focused, and airborne real-time echocardiography precisely locates the myocardial ischemia target location. During the absolute refractory period of electrocardiographic activity, the R wave on the surface electrocardiogram initiates the release of the extracorporeal shock wave (24). The precise mechanisms of CSWT remain to be elucidated. Shock wave therapy has been shown to affect tissue cavitation, which able to produce localized physical forces that may position localized stress on cell membranes (7). This would lead to a variety of biochemical effects including shear stress on cell membranes (25), hyperpolarization and Ras activation (26), an increase in nitric oxide synthesis (27), an up-regulation of vascular endothelial growth factor (VEGF), its receptor Flt-1 and PGF (7, 28), in addition to an enhanced expression of stromal-derived factor-1 (29). Another potential cellular mechanism may involve the recruitment of progenitor cells to the site of the ischemia undergoing CWST (30, 31). In light of these, the positive benefits of CWST are likely the result of several angiogenic mechanisms (7).

The following were this study’s primary conclusions: (1) CSWT enhances left ventricular function, as evidenced by the LVEF and LVEDD; (2) CSWT alleviates angina pectoris symptoms, as evidenced by the decline in CCS class and nitrate consumption; (3) CSWT enhances cardiac function, as evidenced by the decline in NYHA class and the improvement in 6MWT; and (4) CSWT may enhance myocardial viability, as evidenced by the decrease of the score of myocardial perfusion and metabolism imaging.

These findings were consistent with previous studies. Burneikaite et al. (31) included 22 studies and results confirmed that in the majority of published CSWT studies, nitroglycerine consumption and angina frequency decreased, CCS class, SAQ scores and NYHA class improved, myocardial perfusion and exercise capacity increased significantly. And most benefits could be observed as early as in the first month, suggesting the contribution of an early local vasodilating effect of CSWT (31). Wang et al. (32) included 14 studies and found the same results, CSWT improved heart failure condition (supported by the decrease of NYHA class and the improvement of 6MWT and LVEF), relieved the symptom of angina pectoris (supported by the decrease of CCS class and nitroglycerin dosage and the advance of SAQ score), and improved myocardial viability (supported by the lower score of myocardial perfusion and metabolism imaging). In a meta-analysis published in 2020, Yang et al. (24) included 26 studies that found CSWT could improve cardiac function (supported by 6MWT, NYHA class), left ventricular function (supported by LVEF and LVEDD), and relieve angina symptoms (supported by CCS and SAQ score) in CAD patients.

In contrast to the result of these studies, we found a greater heterogeneity and sensitivity of SAQ scores in the included studies. The association of CSWT with improvement in SAQ scores was observed only after the selective exclusion of 1 study. As for the large difference in SAQ scores between the two groups after the experiment, we could not judge the authenticity of the data in this study (21). Besides, our study also did not find significant improvements in exercise duration from CSWT in the studies included in the analysis, and there was a large heterogeneity of studies. We believe this result may be due to differences in the methodologies used to test exercise endurance across these studies. The duration of patients in a study was generally long, and the testing method was not carefully described in the article (15). Moreover, this study is a cohort study and may have methodological differences from other studies.

This study found that the size of the studies included in the current study on CSWT for IHD patients was still small and most were single center. Additionally, the control group’s design was still insufficient. Most randomized trials were rated as having a high risk of bias in terms of attribution, calculating sample sizes, participant blinding, and outcome evaluation. This is due to varied methodological characteristics, flawed designs, or inadequate analyses. In order to more thoroughly assess the clinical function of CSWT in patients with IHD, more rigorous research will be required in the future.

This meta-analysis incorporates the most recent published studies and analyzes a variety of clinical information and is an update on previous studies. The results of this study were different from those of previous studies and provide reference for clinical work. However, some limitations remain. First of all, several of the included research had rather small sample sizes, and the analysis’s relatively narrow population of qualifying studies might have reduced its ability to draw conclusions with sufficient precision. Second, the higher risk of detection and performance bias may diminish the influence of the evidence. Confidentiality of participants or health care professionals may be difficult in CSWT programs, and sham stimulation was used in fewer studies. There are also studies that do not explicitly describe the interventions in the control group. Finally, in addition to RCT studies, other forms were included for analysis. These studies had a control group and did not compare themselves before and after thus these were included in the analysis. This is a limitation of this study, and future studies should fully include RCT studies to improve the quality of the analysis.

## 5. Conclusion

This meta-analysis and systematic review with IHD patients shows consistent evidence that CSWT could improve left ventricular function, relieve the symptom of angina pectoris and improve cardiac function and myocardial viability. However, the quality of the literature is uneven. To verify these findings, more RCT studies with bigger sample numbers and higher methodological standards are required in the future.

## Author contributions

CF conceived and designed research. YM and PS jointly screened the literature in the database. XW reviewed the included literature. XW and YM performed the statistical analysis and wrote the original manuscript. XW and PS interpreted the data. MG revised the original manuscript, reformatted the table, and reviewed the final manuscript. XW and MG reviewed the final data. All authors agreed to submit to the current manuscript, gave final approval of the version to be published, and agreed to be accountable for all aspects of the work.
